# "Per cell" normalization method for mRNA measurement by quantitative PCR and microarrays

**DOI:** 10.1186/1471-2164-7-64

**Published:** 2006-03-29

**Authors:** Jun Kanno, Ken-ichi Aisaki, Katsuhide Igarashi, Noriyuki Nakatsu, Atsushi Ono, Yukio Kodama, Taku Nagao

**Affiliations:** 1 Division of Cellular and Molecular Toxicology, National Institute of Health Sciences, 1-18-1, Kamiyoga, Setagaya-ku, Tokyo 158-8501, Japan; 2 President, National Institute of Health Sciences, 1-18-1, Kamiyoga, Setagaya-ku, Tokyo 158-8501, Japan

## Abstract

**Background:**

Transcriptome data from quantitative PCR (Q-PCR) and DNA microarrays are typically obtained from a fixed amount of RNA collected per sample. Therefore, variations in tissue cellularity and RNA yield across samples in an experimental series compromise accurate determination of the absolute level of each mRNA species per cell in any sample. Since mRNAs are copied from genomic DNA, the simplest way to express mRNA level would be as copy number per template DNA, or more practically, as copy number per cell.

**Results:**

Here we report a method (designated the "Percellome" method) for normalizing the expression of mRNA values in biological samples. It provides a "per cell" readout in mRNA copy number and is applicable to both quantitative PCR (Q-PCR) and DNA microarray studies. The genomic DNA content of each sample homogenate was measured from a small aliquot to derive the number of cells in the sample. A cocktail of five external spike RNAs admixed in a dose-graded manner (dose-graded spike cocktail; GSC) was prepared and added to each homogenate in proportion to its DNA content. In this way, the spike mRNAs represented absolute copy numbers per cell in the sample. The signals from the five spike mRNAs were used as a dose-response standard curve for each sample, enabling us to convert all the signals measured to copy numbers per cell in an expression profile-independent manner. A series of samples was measured by Q-PCR and Affymetrix GeneChip microarrays using this Percellome method, and the results showed up to 90 % concordance.

**Conclusion:**

Percellome data can be compared directly among samples and among different studies, and between different platforms, without further normalization. Therefore, "percellome" normalization can serve as a standard method for exchanging and comparing data across different platforms and among different laboratories.

## Background

Normalization of gene expression data between different samples generated in the same laboratory using a single platform, and/or generated in different geographical regions using multiple platforms, is central to the establishment of a reliable reference database for toxicogenomics and pharmacogenomics. Transforming expression data into a "per cell" database is an effective way of normalizing expression data across samples and platforms. However, transcriptome data from the quantitative PCR (Q-PCR) and DNA microarray analyses currently deposited in the database are related to a fixed amount of RNA collected per sample. Variations in RNA yield across samples in an experimental series compromise accurate determination of the absolute level of each mRNA species per cell in any sample. Normalization against housekeeping genes for PCRs, and global normalization of ratiometric data for microarrays, is typically performed to account for this informational loss. Additional methods, such as the use of external mRNA spikes, reportedly improve the quality of data from microarray systems. For example, Holstege et al. [1] described a spike method against total RNA, based on their finding that the yields of total RNA from wild type and mutant cells were very similar. Hill et al. [2] reported a spike method against total RNA for normalizing hybridization data such that the sensitivities of individual arrays could be compared. Lee et al. [3] demonstrated that "housekeeping genes" cannot be used as a reference control, and van de Peppel et al. [4] described a normalization method of mRNA against total RNA using an external spike mixture. To achieve satisfactory performance they used multiple graded doses of external spikes, covering a wide range of expression, in order to align the ratiometric data by Lowess normalization [5]. Hekstra et al. [6] presented a method for calculating the final cRNA concentration in a hybridization solution. Sterrenburg et al. [7] and Dudley et al. [8] reported the use of common reference control samples for two-color microarray analyses of the human and yeast genomes, respectively. These are pools of antisense oligo sequences against all sense oligos present on the microarray. Instead of antisense oligos, Talaat et al. [9] used genomic DNA as a common reference control in studies of *E. coli*. Statistical approaches have been proposed for ratiometric data to improve inter-microarray variations, especially of non-linear relations [10]. However, because control samples may differ among studies, ratiometric data cannot easily be compared across multiple studies unless a common reference, such as a mixture of all antisense counterparts of spotted sense sequences is used [7-9]. Nevertheless, as long as the normalization is calibrated to total RNA, variations in total RNA profile cannot be effectively cancelled out. Although some of these reports share the idea that "absolute expression" and "transcripts per cell" should entail robust normalization, further practical development to enable universal application has been awaited.

Here, we report a method for normalizing expression data across samples and methods to the cell number of each sample, using the DNA content as indicator. This normalization method is independent of the gene expression profile of the sample, and may contribute to transcriptome studies as a common standard for data comparison and interchange.

## Results

### Dose-response linearity of the measurement system as a basis for the Percellome method

**Figure 1:**
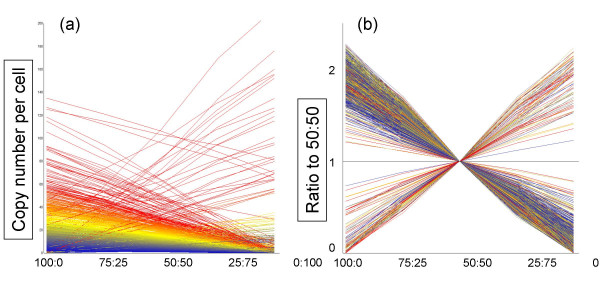
**Dose-response linearity check by LBM**. Dose-response linearity of the Affymetrix GeneChip by the LBM (liver-brain mix) sample set. Five samples, i.e. mixtures of mouse liver and brain at ratios of 100:0, 75:25, 50:50, 25:75 and 0:100, were spiked with GSC and measured by Affymetrix GeneChips Mouse430-2. Signals were normalized by the Percellome method as described in the text. Line graphs are in (a) copy numbers and (b) ratio to 50:50 sample for the top 1,000 probe sets with coefficient of correlation (R^2^) closest to 1 among those having 1 copy or more per cell in the 50:50 sample (19,979 probe sets out of 45,101). The number of probe sets with R^2 ^> 0.950 was 8,655, and R^2 ^> 0.900 was 11,719.

The fidelity of transcript detection is the key to this "per cell" based normalization method, which generates transcriptome data in "mRNA copy numbers per cell". The Q-PCR system was tested by serially diluting samples to confirm the linear relationship between Ct values and the log of sample mRNA concentration (data not shown). High density oligonucleotide microarrays from Affymetrix [11] were used in our experiments. We tested the linearity of the Affymetrix GeneChips using a set of five samples made of mixtures of liver and brain in ratios of 100:0, 75:25, 50:50, 25:75, and 0:100 (designated "LBM" for liver-brain mix). The results showed a linear relationship (R^2 ^> 0.90) between fluorescence intensity and input for a sufficient proportion of probe sets, i.e. about 37% of the probe sets in the older MG-U74v2 and 70% in the newest Mouse Genome 430 2.0 GeneChip were above the detection level (approximately one copy per cell) in the 50:50 sample (Figure 1) [see Additional files 1 and 2].

Dose-response linearity alone is not sufficient to generate true mRNA copy numbers. An important additional requirement is that the ratio of signal intensity to mRNA copy number should be equal among all GeneChip probe sets of mRNAs and PCR primers. The Q-PCR primer sets were designed to perform at similar amplification rates to minimize differences between amplicons. The melting temperature was set between 60° and 65°C with a product size of approximately 100 base pairs using an algorithm (nearest neighbor method, TAKARA BIO Inc., Japan), and the amplification co-efficiency (E) was set within the range 0.9 ± 0.1 (E = 2^{-(1/slope)}-1 on a plot of log2 (template) against Ct value). For the GeneChip system, the signal/copy performance of each probe set depended on the strategy of designing the probes to keep the hybridization constant/melting temperature within a narrow range, ensuring that the dose-response performances of the probe sets were similar (cf. ). Failing this, any differences should at least be kept constant within the same make/version of the GeneChip. Taking into consideration the biases that lead to imperfections in estimating absolute copy numbers in each gene/probe set, we developed normalization methods to set up a common scale for Q-PCR and Affymetrix GeneChip systems.

### The grade-dosed spike cocktail (GSC) and the "spike factor" for the Percellome method

**Figure 2:**
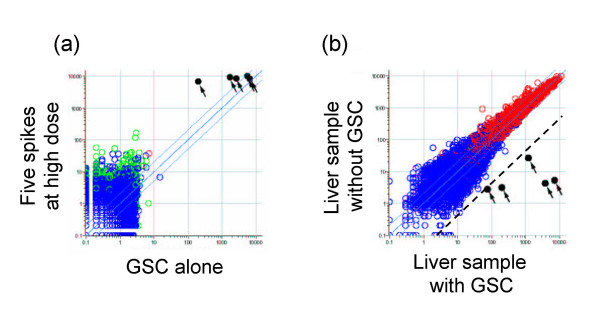
**Cross-hybridization of GSC**. Cross-hybridization of the GSC spike mRNAs to Affymetrix GeneChip. (a) A scatter plot of a blank sample with the GSC (horizontal axis) and a blank with the five spike RNAs at a high dosage (vertical axis) measured by MG-U74v2A GeneChips (raw values generated by Affymetrix MAS 5.0 software). The five spikes are indicated by black dots with arrows. Signals of the murine probe sets were below 20 on the horizontal axis, indicating negligible cross-hybridization of GSC spike mRNAs to the murine probe sets. (b) A scatter plot of a liver sample with GSC (horizontal axis) and without GSC (vertical axis) measured by MG-U74v2A GeneChips. The five spikes are again indicated by black dots with arrows. The dotted line is the 1/25 fold (4%) line. Cross-hybridization of mouse liver mRNAs to the GSC signals was considered negligible (less than 4%).

A set of external spike mRNAs was used to transfer the measurement of cell number in the sample (as reflected by its DNA content) to transcriptome analysis. For the spikes, we utilized five *Bacillus subtilis *mRNAs that were left open for users in the Affymetrix GeneChip series. The extent to which the *Bacillus *RNAs cross-hybridized with other probe sets was checked for the Affymetrix GeneChip system. The GSC was applied to Murine Genome U74Av2 Array (MG-U74v2) GeneChips with or without a liver sample. As shown in Figure 2, cross-hybridization between *Bacillus *RNAs and the murine gene probe sets was negligible [see Additional files 3 and 4]. Mouse Genome 430 2.0 Array (Mouse430-2), Mouse Expression Arrays 430A (MOE430A) and B (MOE430B), Rat Expression Array 230A (RAE230A), *Xenopus laevis *Genome Array and Human Genome U95Av2 (HG-U95Av2) and U133A (HG-U133A) Arrays sharing the same probe sets for these spike mRNAs showed no sign of cross-hybridization with the *Bacillus *probes (data not shown).

**Figure 3:**
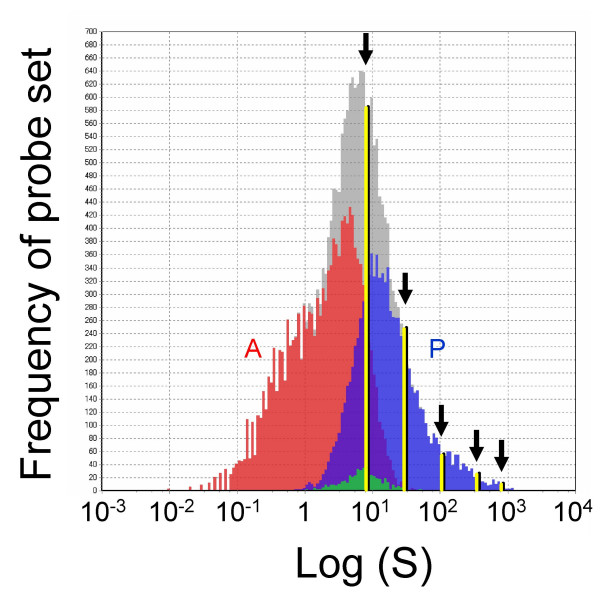
**Positioning of GSC spike mRNAs in Affymetrix GeneChip dose-response range**. A frequency histogram of the probe sets of Affymetrix GeneChip Mouse430-2 is shown. The histogram for all probe sets (gray) shows near-normal distribution. Blue columns are the "present" calls (P), red columns "absent" calls (A) and green "marginal" calls. The five yellow lines indicate the positions of the GSC spike mRNAs that are chosen to cover the "present" call range by a proper "spike factor".

We prepared a cocktail containing *in vitro *transcribed *Bacillus *mRNAs in threefold concentration steps, i.e. 777.6 pM (for AFFX-ThrX-3_at), 259.4 pM (for AFFX-LysX-3_at), 86.4 pM (for AFFX-PheX-3_at), 28.8 pM (for AFFX-DapX-3_at) and 9.6 pM (for AFFX-TrpnX-3_at). By referring to the amount of DNA in a diploid cell and employing a "spike factor" determined by the ratio of total RNA to genomic DNA in a tissue type (Table 1), the spike mRNAs were calculated to correspond to 468.1, 156.0, 52.0, 17.3 and 5.8 copies per cell (diploid), respectively, for the mouse liver samples (spike factor = 0.2). The ratio of mRNAs in the cocktail is empirically chosen depending on the linear range of the measurement system and the available number of spikes. Here, we set the ratio to three to cover the "present" call probe sets of the Affymetrix GeneChip system (Figure 3).

**Table 1:** The spike factors for various organs/tissues

Species	Organ/Tissue (adult, unless otherwise noted)	Spike Factor	total RNA/genomic DNA	SD
Mouse	Liver	0.2	211	46
Mouse	Lung	0.02	22	4
Mouse	Heart	0.05	-	-
Mouse	Thymus	0.01	8	2
Mouse	Colon Epitherium	0.05	105	30
Mouse	Kidney	0.1	-	-
Mouse	Brain	0.1	-	-
Mouse	Suprachiasmatic nucleus (SCN)	0.1	-	-
Mouse	Hypothalamus	0.1	63	4
Mouse	Pituitary	0.1	52	8
Mouse	Ovary	0.02	35	4
Mouse	Uterus	0.02	42	12
Mouse	Vagina	0.02	81	38
Mouse	Testis	0.15	56	7
Mouse	Epididymis	0.07	53	16
Mouse	Bone marrow	0.02	14	3
Mouse	Spleen	0.02	-	-
Mouse	Whole Embryo	0.15	97	36
Mouse	Fetal Telencephalon E10.5–16.5	0.1	48	9
Mouse	Neurosphere (E11.5–14.5)	0.03	42	10
Mouse	E9.5 embryo heart	0.15	58	15
Mouse	cell lines	0.2	-	-
Rat	Liver	0.2	-	-
Rat	Kidney	0.2	-	-
Rat	Uterus	0.04	56	5
Rat	Ovary	0.04	56	9
Human	Cancer Cell Lines	0.2	116	26
Xenopus	liver	0.03	-	-
Xenopus	embryo	0.15	-	-

**Figure 4:**
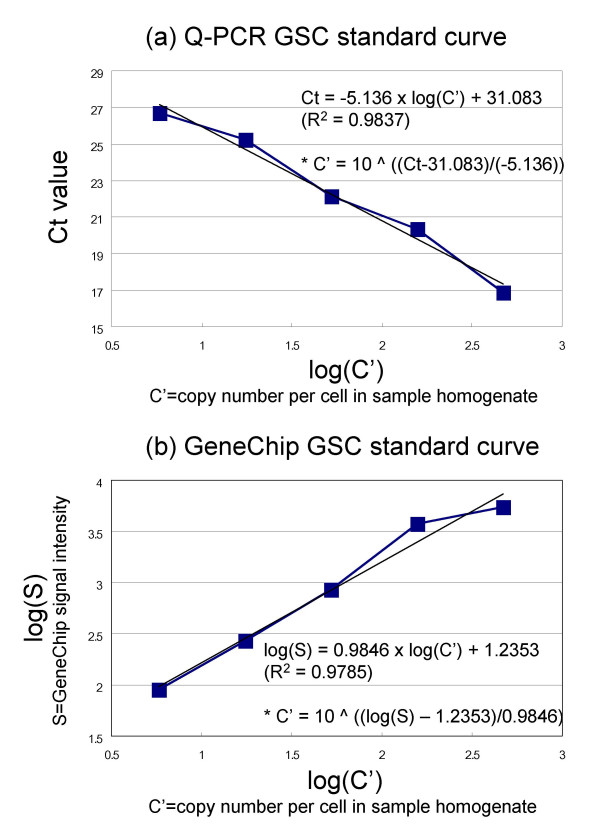
**The dose-response linearity of the GSC spikes in Q-PCR and the Affymetrix GeneChip array system**. Linear relationships are shown between (a) the Q-PCR Ct values and log of copy number (log (C')), and (b) the GeneChip log signal intensity (log(S)) and log of copy number (log (C')) of the GSC mRNAs. The regression functions were obtained by the least squares method. The inverse functions (*) were further used to generate the copy numbers of all other genes/probe sets for Percellome normalization.

We tested this grade-dosed spike cocktail (GSC) by Q-PCR and confirmed that the Ct values of the spike mRNAs were linearly related to the log concentrations (cf. Figure 4a), i.e. could be expressed as

Ct = αlog C + β     {1}

The GSC was also tested by the GeneChip system and it was confirmed that the log of the spike mRNA signal intensities was linearly related to the log of their concentrations (cf. Figure 4b),

log S = γlog C + δ     {2}

The linear relationship between the Ct values (Ct) and the log of RNA concentration (log C) was reasonable given the definition of Ct values (derived from the number of PCR cycles, i.e. doubling processes). The linear relationship between the log of GeneChip signal intensity (log S) and the log of RNA concentration (log C) was rationalized by the near-normal distribution of log S over all transcripts (cf. Figure 3).

### Calculation of copy numbers of all genes/probe sets per cell

As described above, using a combination of DNA content and the spike factor of the sample, the GSC spike mRNAs become direct indicators of the copy numbers (C') per cell. When the samples were measured by Q-PCR or GeneChip analysis, the five GSC spike signals in each sample should obey function {1} for Q-PCR and function {2} for GeneChip with a good linearity. If the observed linearity was poor, a series of quality controls was performed and the measurement repeated. The coefficients of the functions were determined for each sample by the least squares method. Under the assumption that all genes/probe sets share the same signal/copy relationship, signal data for all genes/probe sets were fitted to the functions {1'} or {2'}, which are the individualized functions of {1} and {2} for each sample measurement (i).

Ct = αi log(C') + βi     {1'}

Log (S) = γi log(C') + δi    {2'}

(i = sample measurement no.)

The Q-PCR Ct values (Ct) and microarray signal values (S) of all mRNA species in the sample (i) are converted to copy numbers per cell (C') by the inverses of functions {1'} and {2'}, i.e. {3} and {4} below:

C' = B^((Ct-βi)/αi)     {3}

for Q-PCR (Figure 4a).;

C' = B^((logS-γi)/δi)     {4}

for GeneChips (Figure 4b),

where B is the logarithmic base used in {1} and {2} (see Materials and Methods for details).

### Real world performance of the Percellome method

**Figure 5:**
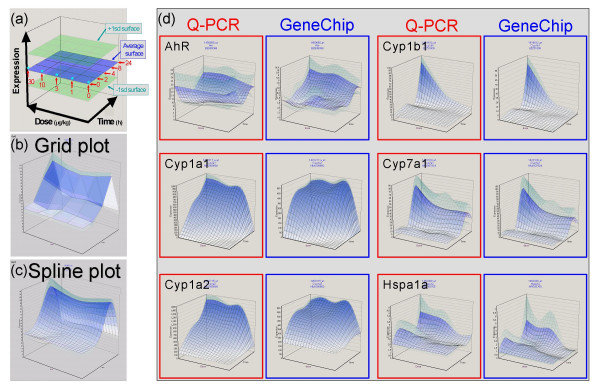
**Correspondence between Q-PCR and GeneChip data**. Sixty male C57BL/6 mice were divided into 20 groups of 3 mice each. 2,3,7,8-tetrachlorodibenzodioxin (TCDD) was administered once orally at doses of 0, 1, 3, 10 and 30μg/kg, and the liver was sampled 2, 4, 8 and 24 h after administration. The liver transcriptome was measured by the Affymetrix Mouse430-2 GeneChip. For Q-PCR, nineteen primary pairs were prepared and the Ct values of the same 60 liver samples were measured (19 genes and 5 spikes in duplicate, using a 96-well plate for 2 samples, total 30 plates). The Percellome data were plotted on to 3-dimensional graphs for average, +1sd, and – 1sd surfaces as shown in (a). The scale of expression (vertical axis) is the copy number per cell. The 0 h data (*) are copied from the 2 h/dose 0 point for better visualization of the changes after 2 h. The surfaces are demonstrated as a grid plot (b) where the grid points indicate one treatment group (n = 3), and a smoothened spline surface plot (c) for easier 3D recognition ((b), (c): Gys2 (glycogen synthase 2, 1424815_ at) showing a typical circadian pattern. (d) the smoothened plots of 6 representative genes/ probe sets generated by Q-PCR (red) and GeneChip (blue). AhR (arylhydrocarbon receptor, 1450695_at)  showed imperfect correspondence. Cyp1a1 (cytochrome P450, family 1, subfamily a, polypeptide 1, 1422217_a_at) and Cyp1a2 (1450715_at) showed good correlations between Q-PCR and GeneChip except for the saturation in GeneChips above c. 400 copies per cell. Cyp1b1 (1416612_at) and Cyp7a1 (1422100_at) showed good correspondence. Hspa1a (heat shock protein 1A, 1452888_at) showed fair correspondence despite low copy numbers, near the nominal detection limit of the Affymetrix GeneChip system.

The correspondence between Q-PCR and GeneChip was tested using a sample set from 2,3,7,8-tetrachlorodibenzodioxin (TCDD)-treated mice. Sixty male C57BL/6 mice were divided into 20 groups of 3 mice each. TCDD was administered once orally at doses of 0, 1, 3, 10 and 30 μg/kg, and the livers were sampled 2, 4, 8 and 24 h after administration. Nineteen primer pairs were prepared for Q-PCR and the Ct values of the liver transcriptome were measured. The same 60 liver samples were measured using the Affymetrix Mouse430-2 GeneChip [see Additional files 5 through 8 and 9 through 12]. Q-PCR and GeneChip data were normalized against cell number by functions {3} and {4}, respectively. The averages and standard deviations (sd) of each group (n = 3) were calculated and plotted as three layers of isoborograms on to 5 × 4 matrix three-dimensional graphs (Figure 5). Together with another sample set (data not shown), a total of thirty-six primer pairs were compared, and there was a correlation of up to 90% between the Q-PCR and GeneChip surfaces. It is notable that not only the average surfaces but also the +1sd and -1sd surfaces corresponded closely in shape and size. We infer that the differences resulted mainly from biological variations among the three animals in each experimental group rather than from measurement error (cf. Figure 7).

**Figure 6:**
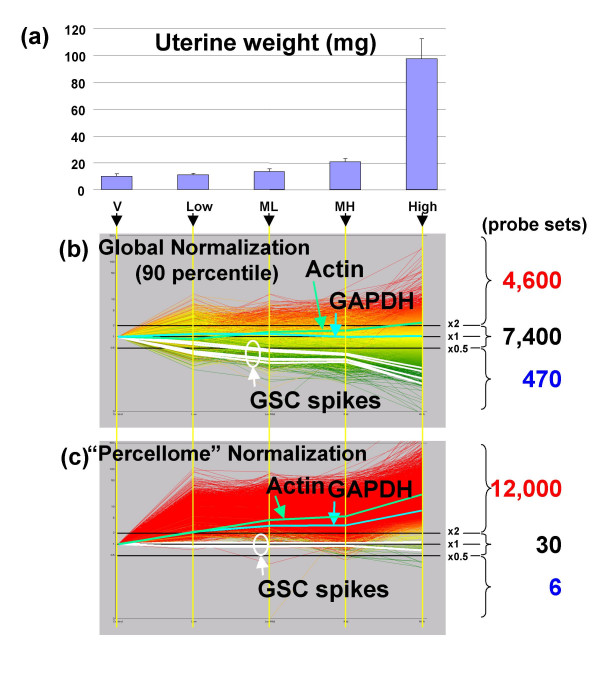
**Uterotrophic response of ovariectomized female mice by an estrogenic test compound**.(a) Shows the uterine weight, which increases in a dose-dependent manner; V, vehicle control; Low, low dose; ML, medium-low dose; MH, medium-high dose; High, high dose group. (b) Shows the line display of uterine gene expression (Affymetrix MG-U74v2 A GeneChips) normalized by global normalization (90 percentile), and (c) by the Percellome normalization. Averages of three samples per group were visualized (by K. A.). The five white lines are the GSC mRNAs. The green and blue lines are actin (AFFX-b-ActinMur/M12481_3_at) and GAPDH (glyceraldehyde-3-phosphate dehydrogenase, AFFX- GapdhMur/M32599_3_at), respectively. By global normalization, 7,400 probe sets remained unchanged and 4,600 probe sets increased more than two-fold in the H group compared to the V group, whereas almost all probe sets measured had increased. It is noted that housekeeping genes such as actin and GAPDH are significantly induced on a per cell basis.

**Figure 7:**
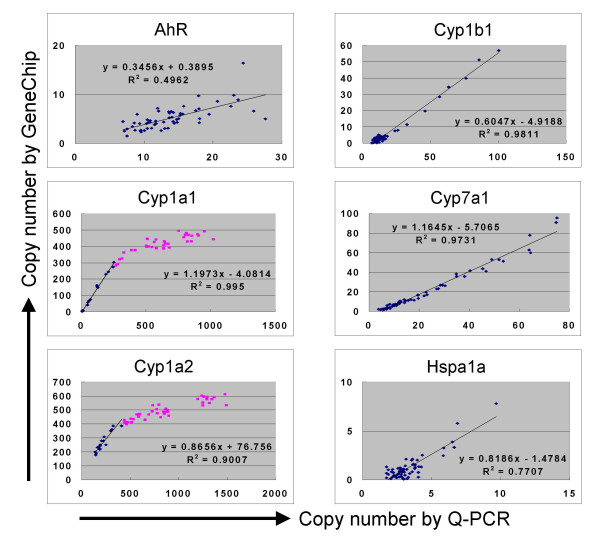
**Conversion functions between Q-PCR and GeneChip**. The data shown in Figure 5 as 3D surfaces are shown as a scatter plot (60 plots). The regression function can be used to convert Q-PCR to GeneChip and vice versa, with a level of certainty indicated by coefficient of correlation. It is noted that Cyp1a1 and Cyp1a2 became saturated above about 400 copies per cell in GeneChip system (indicated in pink plots). Cyp7a1 showed high linearity, indicating that the variation shown by the split +1sd and -1sd surfaces in Figure 5 reflected biological (animal) variation, not measurement errors.

An important feature of Percellome normalization is its independence from the overall expression profile of the sample. When gene expression profiles differ among samples, Percellome normalization produces a robust transcriptome that is different from total-RNA dependent global normalization. As an example, Figure 6 shows the results of an experiment on the uterotrophic response of ovariectomized mice to estrogen treatment [12] [see Additional files 13 and 14]. The uteri of the vehicle control are atrophic because the ovaries, the source of intrinsic estrogens, are absent. The uteri of the treated groups are hypertrophic owing to estrogenic stimulus from the test compound administered. Global normalization (90 percentile) between the vehicle control group and the high-dose (1,000 mg/kg) group indicated that 4,600 of 12,000 probe sets showed 2-fold or greater increase, 470 were reduced by 0.5 or less, and 7,400 remained between these extremes. In contrast, analysis of Percellome-normalized data revealed that almost all the 12,000 probe sets showed a 2-fold or greater increase, including actin, GAPDH and other housekeeping genes. The hypertrophic tissues, consisting of cells with abundant cytoplasm, provide convincing evidence for the increases in various cellular components including housekeeping gene products.

Another important feature of Percellome normalization is the commonality of the expression scale across platforms. Batch conversion can be performed between results obtained from different platforms when the data are generated by the Percellome method. A practical strategy for such normalization is to prepare a set of samples from a target organ of interest with differences in gene expression, and measure them once by each platform. Data conversion functions with good linear dose-response relationships can be obtained individually for those genes/probe sets that are measured by both platforms (Figure 7).

## Discussion

We have developed a novel method for normalizing mRNA expression values to sample cell numbers by adding external spike mRNAs to the sample in proportion to the genomic DNA concentration. For non-diploid or aneuploid samples, an average DNA content per cell should be determined beforehand for accurate adjustment. When there is significant DNA synthesis, a similar adjustment should be considered.

The smallest sample to which we have successfully applied the direct DNA quantification method with sufficient reproducibility is the 6.75 dpc (days post coitus) mouse embryo which consists of approximately 5,000 cells. This sample size is also approximately the lower limit for double amplification protocol to obtain sufficient amount of RNA for Affymetrix GeneChip measurement (cf. .) High-resolution technology such as laser-capture microdissection (LCM) has become popular and the average sample size analyzed is getting smaller. An alternative method for LCM samples is to count the cell number in the course of microdissection. Although we have not yet applied Percellome method to LCM samples, we have applied the alternative method to cell culture samples to gain Percellome data. Stereological and statistical calculations should become available to correct the number of partially sectioned cells in the LCM samples. Another issue for small samples is the yield of RNA. Approximately 30 ng of total RNA is retrieved from a single 6.75 dpc mouse embryo. This amount is sufficient for a double amplification protocol (DA) to prepare enough RNA for an Affymetrix GeneChip measurement. An inherent problem with the DA data is that the gene expression profile differs from that of the default single amplification protocol (SA). Consequently the DA percellome data differ from that of SA as if they were produced by a different platform. To bridge the difference, we applied the procedure that was used for data conversion between Q-PCR and GeneChip (cf. Figure 7). A set of spiked-in standard samples including the LBM sample set (of sufficient concentration) were measured by the SA protocol and diluted versions to the limit measured by the DA protocol. These data provided us with information about whether DA was successful as a whole (by comparing 5' signal to 3' signals of selected probe sets) and which probe sets were properly amplified by DA (by checking the linearity of the diluted LBM data). For those probe sets that proved to be linearly amplified, conversion functions between DA and SA were generated. These details, along with embryo expression data will be published elsewhere.

Figures 5 and 7 indicate a close correspondence between the data generated by Q-PCR and GeneChip analyses. Since each of the 60 samples was normalized individually against each GSC signal, the high similarity between the two platforms indicates the robustness and stability of this spike system (cf. Figure 7, Cyp7a1 data). Although more spikes could potentially increase the accuracy of normalization, our experience is that five spikes are practically sufficient for covering the detection range of GeneChip microarrays and Q-PCR, as long as they are used in combination with the "spike factor". The overall benefits of using a minimum number of external spikes include lower probability of cross-hybridization, a reduced number of wells and spots occupied by the spikes in the Q-PCR plates and small scale microarrays, and less effort in preparation, QC and supply.

The Percellome data can be truly absolute when all mRNA measurements including GSC spikes are strictly proportional to the original copy numbers in the sample homogenate. As noted earlier, this condition is not guaranteed by any platform despite linearity of response. Therefore, the Percellome-normalized values have some biases for each primer pair/probe set, depending on the steepness of the dose-response curves. An advantage of Percellome normalization is that, as long as such biases are consistently reproduced within a platform, the data can be compared directly among samples/studies on a common scale. Consequently, when a true value is obtained by any other measure, all the data obtained in the past can be simultaneously batch-converted to the true values.

This batch-conversion strategy can be extended to data conversion between different versions and different platforms, as long as the data are generated in copy numbers "per cell". We have shown an example between Affymetrix GeneChip and Q-PCR for limited numbers of probe sets (cf. Figure 7). Custom microarrays that accept our GSC for Percellome normalization are in preparation by Agilent Technologies (single color) and GE Healthcare (CodeLink Bioarray).

Another important contribution of Percellome analysis is in the area of archived data in private and public domains. Firstly, Percellome data are the result of a simple linear transformation of the raw microarray data; preserving the distribution and order of the probe set data. Therefore, parametric or non-parametric methods should be able to align the data distribution and generate estimates of mRNA copy number of the non-spiked archival samples. Any archival samples that are re-measurable by Percellome method will greatly increase the accuracy of estimation. Secondly, percellome can provide appropriate bridging information between old and new versions of Affymetrix GeneChips, such as human HU-95 and HU-133, murine MU-74v2 and MOE430 series. This should also facilitate comparisons between newly generated and archived data.

The Percellome method was developed for a large-scale toxicogenomics project [13] using the Affymetrix GeneChip system. It was intended to compile a very large-scale database of comprehensive gene expression profiles in response to various chemicals from a series of experiments conducted over an extended time period. However, the method also proved to be useful for small-scale platforms such as 96 well plate-based Q-PCRs as shown above, and probably for small-scale targeted microarrays. In both cases, highly inducible or highly transcribed genes are likely to be selected. Therefore, the expression profiles may differ significantly among samples such that profile-dependent normalization (e.g. global normalization) may not be applicable. In such cases, the profile-independent nature of the Percellome method provides a robust normalization.

To demonstrate the profile-independence of the Percellome method, we chose an extreme case – the uterotrophic response assay (cf. Figure 6). The treated uteri were composed of hypertrophic cells with abundant cytoplasm whereas the untreated uteri were composed of hypoplastic cells with scant cytoplasm. This indicates that the uteri of untreated ovariectomized mice were quiescent, and that a majority of the inducible genes were probably transcriptionally inactive. Therefore, the identification of most genes as being induced by 2-fold or greater is reasonable and expected. In most *in vivo *experiments, the gene profiles of the samples are much more similar. However, there is always a set of genes that is found to be "increased" when analyzed on a "per one cell" basis that are declared to be "decreased" by global type normalization, or vice versa. Such increase/decrease calls made by the global type normalization can differ according to the normalization parameters. In both cases, the Percellome method can inform the researcher how much the expression profiles are distorted by the treatment, such as in the case of the uterotrophic assay. We also note that *in vitro *experiments such as cell-based studies tend to generate data similar to that of uterotrophic experiment.

## Conclusion

Percellome data can be compared directly among samples and among different studies, and between different platforms, without further normalization. Therefore, "percellome" normalization can serve as a standard method for exchanging and comparing data across different platforms and among different laboratories. We hope that the Percellome method will contribute to transcriptome-based studies by facilitating data exchanges among laboratories.

## Methods

### Animal experiments

C57BL/6 Cr Slc (SLC, Hamamatsu, Japan) mice maintained in a barrier system with a 12 h photoperiod were used in this study. For the liver transcriptome experiments, twelve week-old male mice were given a single dose of the test compound by oral gavage, and the liver was sampled at 2, 4, 8 and 24 h post-gavage. For the uterotrophic experiment, 6 week old female mice were ovariectomized 14 days prior to the 7 day repeated subcutaneous injection of a test compound [12]. Animals were euthanized by exsanguination under ether anesthesia and the target organs were excised into ice-cooled plastic dishes. Tissue blocks weighing 30 to 60 mg were placed in an RNase-free 2 ml plastic tube (Eppendorf GmbH., Germany) and soaked in RNAlater (Ambion Inc., TX) within 3 min of the beginning of anesthesia. Three animals per treatment group were used and individually subjected to transcriptome measurement.

### Sample homogenate preparation

The tissue blocks soaked in RNAlater were kept overnight at 4°C or until use. RNAlater was replaced in the 2 ml plastic tube with 1.0 ml of RLT buffer (Qiagen GmbH., Germany), and the tissue was homogenized by adding a 5 mm diameter Zirconium bead (Funakoshi, Japan) and shaking with a MixerMill 300 (Qiagen GmbH., Germany) at a speed of 20 Hz for 5 min (only the outermost row of the shaker box was used).

#### Direct DNA quantitation

Three separate 10 μl aliquots were taken from each sample homogenate to another tube and mixed thoroughly. A final 10 μl aliquot therefrom was treated with DNAse-free RNase A (Nippon Gene Inc., Japan) for 30 min at 37°C, followed by Proteinase K (Roche Diagnostics GmbH., Germany) for 3 h at 55°C in 1.5 ml capped tubes. The aliquot was transferred to a 96-well black plate. PicoGreen fluorescent dye (Molecular Probes Inc., USA) was added to each well, shaken for 10 seconds four times and then incubated for 2 min at 30°C. The DNA concentration was measured using a 96 well fluorescence plate reader with excitation at 485 nm and emission at 538 nm. λ phage DNA (PicoGreen Kit, Molecular Probes Inc., USA) was used as standard. Measurement by this PicoGreen method and the standard phenol extraction method correlated well (coefficient of correlation = 0.97, data not shown). The smallest sample size for reproducible and reliable DNA quantitation is about 5,000 cells that corresponds to a 6.75 dpc mouse embryo.

### The grade-dosed spike cocktail (GSC)

The following five *Bacillus subtilis *RNA sequences were selected from the gene list of Affymetrix GeneChip arrays (AFFX-ThrX-3_at, AFFX-LysX-3_at, AFFX-PheX-3_at, AFFX-DapX-3_at, and AFFX-TrpnX-3_at) present in the MG-U74v2, RG-U34, HG-U95, HG-U133, RAE230 and MOE430 arrays: thrC, thrB genes corresponding to nucleotides 248–2229 of X04603; lys gene for diaminopimelate decarboxylase corresponding to nucleotides 350–1345 of X17013; pheB, pheA genes corresponding to nucleotides 2017–3334 of M24537, dapB, jojF, jojG genes corresponding to nucleotides 1358–3197 of L38424; TrpE protein, TrpD protein, TrpC protein corresponding to nucleotides 1883–4400 of K01391. The corresponding cDNAs were purchased from ATCC, incorporated into expression vectors, amplified in *E. coli *and transcribed using the MEGAscript kit (Ambion Inc., TX). The mRNA was purified using a MACS mRNA isolation kit (Miltenyi Biotec GmbH., Germany). The concentrations of spike RNAs in the GSC were in threefold steps, from 777.6 pM for AFFX-ThrX-3_at, 259.4 pM for AFFX-LysX-3_at, 86.4 pM for AFFX-PheX-3_at, 28.8 pM for AFFX-DapX-3_at, to 9.6 pM for AFFX-TrpnX-3_at. In general, the ratio depends on the linear range of the measurement system and the available number of spikes.

### Setting of the "spike factor" and addition of GSC to a sample homogenate according to its DNA concentration

The GSC was added to the sample homogenates in proportion to their DNA concentrations, assuming that all cells contain a fixed amount of genomic DNA (g/cell) across samples. The amount of GSC added to each sample G (l) was given as

G = C * v * f     (l),

where C is the DNA concentration (g/l), v(l) is the volume of homogenate further used for RNA extraction and f (l/g) is the "spike factor", which is an adjustment factor to ensure that the sample is properly spiked by the GSC (cf. Figure 3). Spike factors have been pre-determined for various organs/tissues to reflect differences in their total RNA/genomic DNA ratios (cf. Table 1). In this way, five spike mRNA signals can properly cover the linear dose-response range of the platform. In practice, for the Affymetrix GeneChips, the spike factor is set so that the five GSC spikes cover the range of "Present" calls given by the Affymetrix system, which corresponds to approximately 80 to 7000 in raw readouts given by the Affymetrix MAS5.0 software. A raw readout of 10 by the current Affymetrix GeneChip system corresponds to approximately one copy per cell in mouse liver (spike factor = 0.2), whereas in mouse thymus (spike factor = 0.01) it corresponds to approximately 0.05 copy per cell. For Q-PCR, the same spike factor corresponds to Ct values ranging approximately from 17 to 27, which is well within the linear range of Q-PCR (data not shown).

### "Per cell" normalization (Percellome normalization)

Since murine haploid genomic DNA is made of 2.5 × 10^9 ^base pairs and one base pair is approximately 600 Daltons (Da), the haploid genomic DNA weighs 1.5 × 10^12 ^Da, corresponding to

d = 5 × 10^-12 ^(g DNA per diploid cell).

Therefore, the cell number per liter of the sample homogenate (N) is given as

N = C/d (cells/l)

where C is the DNA concentration (g/l).

On the other hand, the copy numbers of GSC RNAs in the homogenate are given as follows:

if Sj (mole/l) (j = 1,2,3,4,5) is the mole concentration of one of the five spike RNAs in the GSC solution and G(l) is the amount of GSC added to each homogenate, the mole concentrations of the spike RNAs in the homogenate (CSj) are given as,

CSj = Sj*C*f (mole/l).

The GSC RNAs in moles per cell (MSj) are given as

MSj = CSj/N

= Sj*C*f/(C/d)

= Sj*f*d (mole/cell)

The copy numbers of the GSC RNAs per cell (NSj) are given as

NSj = MSj*A

= Sj*f*d*A (copies per diploid cell)

where A is Avogadro's number.

As a result, the GSC spikes AFFX-TrpnX-3_at, AFFX-DapX-3_at, AFFX-PheX-3_at, AFFX-LysX-3_at and AFFX-ThrX-3_at correspond approximately to 5.8, 17.3, 52.0, 156.0 and 468.1 copies per cell (per diploid DNA template) for mouse liver sample homogenates, where the spike factor = 0.2. It is our observation that the RNA/DNA ratios are virtually constant across polyploid hepatocytes (data not shown).

For each Q-PCR plate or GeneChip, the coefficients, α, β, γ and δ of functions {1} or {2} are determined from the GSC values using the least-square method. The signal values or Ct values of all the other mRNAs measured are then converted to copy numbers per cell by {3} or {4}, i.e. the inverses of functions {1} or {2}.

### The "LBM" ("liver-brain mix") standard sample

A pair of samples having dissimilar gene expression profiles was chosen to evaluate the linearity of the platform. The pairs chosen were brain and liver for mouse and rat, two distinct cancer cell lines for humans, and adult liver and embryo for *Xenopus laevis*. The sample pairs were processed as described above including addition of the GSC. Two final homogenates were then blended at ratios of 100:0, 75:25, 50:50, 25:75 and 0:100 (based on cell numbers) to make five samples. These five samples were measured by Q-PCR and/or GeneChips (MG-U74v2A, MEA430A, MEA430B, MG430 2.0 (shown in Figure 1), RAE230A, HG-U95A, HG-U133, and Xenopus array).

### Quantitative-PCR

Duplicate homogenate samples were treated with DNaseI (amplification grade, Invitrogen Corp., Carlsbad, CA, USA) for 15 min at room temperature, followed by SuperScript II (Invitrogen) for 50 min at 42°C for reverse transcription. Quantitative real time PCR was performed with an ABI PRISM 7900 HT sequence detection system (Applied Biosystems, Foster City, CA, USA) using SYBR Premix Ex Taq (TAKARA BIO Inc., Japan), with initial denaturation at 95°C for 10 s followed by 45 cycles of 5 s at 95°C and 60 s at 60°C, and Ct values were obtained. Primers for the genes explored in this study were selected from sequences close to the areas of Affymetrix GeneChip probe sets as shown in Table 2.

**Table 2:** Primers for Q-PCR

Gene	Forward	Reverse
AFFX-TrpnX-3_at	TTCTCAGCGTAAAGCAATCCA	GCAAATCCTTTAGTGACCGAATACC
AFFX-DapX-3_at	TCAGCTAACGCTTCCAGACC	GGCCGACAGATTCTGATGACA
AFFX-PheX-3_at	GCCAATGATATGGCAGCTTCTAC	TGCGGCAGCATGACCATTA
AFFX-LysX-3_at	CCGCTTCATGCCACTGAATAC	CCGGTTCGATCCAAATTTCC
AFFX-ThrX-3_at	CCTGCATGAGGATGACGAGA	GGCATCGGCATATGGAAAC
Ahr_1450695_at	CAGAGACCACTGACGGATGAA	AGCCTCTCCGGTAGCAAACA
Cyp1a1_1422217_a_at	TGCTCTTGCCACCTGCTGA	GGAGCACCCTGTTTGTTTCTATG
Cyp1a2_1450715_at	CCTCACTGAATGGCTTCCAC	CGATGGCCGAGTTGTTATTG
Cyp1b1_1416612_at	GCCTCAGGTGTGTTTGATGGA	AGTACAGCCCTGGTGGGAATG
Cyp7a1_1422100_at	TTCTACATGCCCTTTGGATCAG	GGACACTTGGTGTGGCTCTC
Hspa1a_1452388_at	ACCATCGAGGAGGTGGATTAGA	AGGACTTGATTGCAGGACAAAC

### Affymetrix GeneChip measurement

The sample homogenates with GSC added were processed by the Affymetrix Standard protocol. The GeneChips used were MG-U74v2A for the uterotrophic study and Mouse 430-2 for the TCDD study (singlet measurement). The efficiency of *in vitro *transcription (IVT) was monitored by comparing the values of 5' probe sets and 3' probe sets of the control RNAs (AFFX- probe sets) including the GSC (see Quality Control below). The dose-response linearity of the five GSC spikes was checked and samples showing saturation and/or high background were re-measured from either backup tissue samples, an aliquot of homogenate, or a hybridization solution, depending on the nature of the anomaly.

### Quality control

Any external spiking method, including our Percellome method, is valid for high-quality RNA samples. Therefore, the quality of the sample RNA should be carefully monitored. In addition to a common checkup by RNA electrophoresis (including capillary electrophoresis if necessary), OD ratio, and cRNA yield, we monitor the performance of IVT (*in vitro *translation) or amplification. The 3' and 5' probe set data of the spiked-in RNAs and sample RNAs (actin, GAPD and other AFFX- probe sets) that are prepared in Affymetrix GeneChip are compared to monitor the extension of RNA by the IVT process. When both the spiked-in RNAs and the sample RNAs have similar levels of 5' and 3' signals respectively, it is judged that the IVT extension was normally performed. When both spiked-in and sample RNAs have significantly lower 5' signal than 3' signal, it is judged that the IVT extension was abnormal. When only the sample RNAs showed significantly lower 5' signal than 3' signal, it is judged that the IVT extension was normal but the sample RNAs were degraded. When only the spiked-in RNAs showed significantly lower 5' signal than 3' signal, it is judged that the IVT extension was normal but the spiked-in RNAs were degraded (although we have not encountered this situation). In addition, if the degraded sample was spiked-in by the non-degraded spike RNAs and measured by GeneChip, the position of spiked-in RNAs will be offset toward abnormally higher intensity. Together, this battery of checkups considerably increases the ability to detect abnormal events that will affect the reliability of the Percellome method. When any abnormality was found, each step of sample preparation was reevaluated to regain normal data for Percellome normalization.

### The web site for GeneChip data

The GeneChip data are accessible at .

## Authors' contributions

JK drafted the concept of the Percellome method, led the project at a practical level, and drafted the manuscript. KA developed the algorithm for the Percellome calculation and wrote the calculation/visualization programs. KI developed the laboratory protocols for the Percellome procedures to the level of SOP for technicians. NN developed the Percellome Q-PCR protocol and performed the measurements, and helped in analyzing the Percellome data. AO helped develop the algorithm. YK led the animal studies. TN provided advice and led the toxicogenomics project using the Percellome method, to be approved by the Ministry of Health, Labour and Welfare of Japan.

## Supplementary Material

Additional File 1 Excel spreadsheet file containing 15 Affymetrix Mouse 430–2 GeneChip raw data of five LBM samples in triplicate (cf. Figure 1). The column name LBM-100-0-X_Signal indicates the component percentages, i.e. 100% liver 0% brain, and X = 1,2,3 indicates the triplicates. The LBM-100-0-X_Detection column indicates P for present, A for absent and M for marginal calls by Affymetrix MAS 5.0 system. Click here for file

Additional File 2 Excel spreadsheet file containing Percellome data of the same LBM samples, of which raw data is listed in Additional file 1 (cf. Figure 1). Click here for file

Additional File 3 Excel spreadsheet file containing 2 Affymetrix MG-U74v2 raw data of a blank sample with the GSC (horizontal axis of Figure 2a) and blank with the five spike RNAs at a high dosage (vertical axis of Figure 2a). Click here for file

Additional File 4 Excel spreadsheet file containing 2 Affymetrix MG-U74v2 raw data of a liver sample with GSC (horizontal axis of Figure 2b) and without GSC (vertical axis of Figure 2b). Click here for file

Additional File 5 (first quarter of a data set consisting of 2 hr, 4 hr, 8 hr, and 24 hr data, divided because of the upload file size limitation)]: an Excel spreadsheet file containing 2 hr data (15 GeneChip data) of the total of 60 Affymetrix Mouse 430-2 GeneChip raw data of the TCDD study consisting of 20 different treatment groups in triplicate (cf. Figure 5). The column name DoseXXX-TimeYY-Z_Signal indicates the dosage and sampling time after TCDD administration in hours, e.g. XXX = 001 indicates 1 microgram/kg group, YY = 02 indicates two hours after administration, and Z = 1,2,3 indicates animal triplicate. The DoseXXX-TimeYY-Z_Detection column indicates P for present, A for absent and M for marginal calls by Affymetrix MAS 5.0 system. Click here for file

Additional File 6 (second quarter of a data set consisting of 2 hr, 4 hr, 8 hr, and 24 hr data, divided because of the upload file size limitation)]: an Excel spreadsheet file containing 4 hr data (15 GeneChip data) of the total of 60 Affymetrix Mouse 430-2 GeneChip raw data of the TCDD study consisting of 20 different treatment groups in triplicate (cf. Figure 5). The column name DoseXXX-TimeYY-Z_Signal indicates the dosage and sampling time after TCDD administration in hours, e.g. XXX = 001 indicates 1 microgram/kg group, YY = 02 indicates two hours after administration, and Z = 1,2,3 indicates animal triplicate. The DoseXXX-TimeYY-Z_Detection column indicates P for present, A for absent and M for marginal calls by Affymetrix MAS 5.0 system. Click here for file

Additional File 7 (third quarter of a data set consisting of 2 hr, 4 hr, 8 hr, and 24 hr data, divided because of the upload file size limitation)]: an Excel spreadsheet file containing 8 hr data (15 GeneChip data) of the total of 60 Affymetrix Mouse 430-2 GeneChip raw data of the TCDD study consisting of 20 different treatment groups in triplicate (cf. Figure 5). The column name DoseXXX-TimeYY-Z_Signal indicates the dosage and sampling time after TCDD administration in hours, e.g. XXX = 001 indicates 1 microgram/kg group, YY = 02 indicates two hours after administration, and Z = 1,2,3 indicates animal triplicate. The DoseXXX-TimeYY-Z_Detection column indicates P for present, A for absent and M for marginal calls by Affymetrix MAS 5.0 system. Click here for file

Additional File 8 (last quarter of a data set consisting of 2 hr, 4 hr, 8 hr, and 24 hr data, divided because of the upload file size limitation)]: an Excel spreadsheet file containing 24 hr data (15 GeneChip data) of the total of 60 Affymetrix Mouse 430-2 GeneChip raw data of the TCDD study consisting of 20 different treatment groups in triplicate (cf. Figure 5). The column name DoseXXX-TimeYY-Z_Signal indicates the dosage and sampling time after TCDD administration in hours, e.g. XXX = 001 indicates 1 microgram/kg group, YY = 02 indicates two hours after administration, and Z = 1,2,3 indicates animal triplicate. The DoseXXX-TimeYY-Z_Detection column indicates P for present, A for absent and M for marginal calls by Affymetrix MAS 5.0 system. Click here for file

Additional File 9 (first quarter of a data set consisting of 2 hr, 4 hr, 8 hr, and 24 hr data, divided because of the upload file size limitation)]: an Excel spreadsheet file containing 2 hr Percellome data (15 sample data) of the 60 samples of the TCDD study (cf. Figure 5), of which corresponding raw data is listed in Additional file 5. Click here for file

Additional File 10 (second quarter of a data set consisting of 2 hr, 4 hr, 8 hr, and 24 hr data, divided because of the upload file size limitation)]: an Excel spreadsheet file containing 4 hr Percellome data (15 sample data) of the 60 samples of the TCDD study (cf. Figure 5), of which corresponding raw data is listed in Additional file 6. Click here for file

Additional File 11 (third quarter of a data set consisting of 2 hr, 4 hr, 8 hr, and 24 hr data, divided because of the upload file size limitation)]: an Excel spreadsheet file containing 8 hr Percellome data (15 sample data) of the 60 samples of the TCDD study (cf. Figure 5), of which corresponding raw data is listed in Additional file 7. Click here for file

Additional File 12 (last quarter of a data set consisting of 2 hr, 4 hr, 8 hr, and 24 hr data, divided because of the upload file size limitation)]: an Excel spreadsheet file containing 24 hr Percellome data (15 sample data) of the 60 samples of the TCDD study (cf. Figure 5), of which corresponding raw data is listed in Additional file 8. Click here for file

Additional File 13 Excel spreadsheet file containing 15 Affymetrix MG-U74v2 A GeneChip raw data of the uterotrophic response study (cf. Figure 6). The column name X-Y_Signal indicates the treatment (V = vehicle, Low = low dose, etc) and animal triplicate (Y = 1,2,3). The X-Y_Detection column indicates P for present, A for absent and M for marginal calls by Affymetrix MAS 5.0 system. Click here for file

Additional File 14 Excel spreadsheet file containing Percellome data of the same 15 samples of the uterotrophic response study (cf. Figure 6), of which raw data is listed in Additional file 13. Click here for file

## References

[B1] Holstege FC, Jennings EG, Wyrick JJ, Lee TI, Hengartner CJ, Green MR, Golub TR, Lander ES, Young RA (1998). Dissecting the regulatory circuitry of a eukaryotic genome. Cell.

[B2] Hill AA, Brown EL, Whitley MZ, Tucker-Kellogg G, Hunter CP, Slonim DK (2001). Evaluation of normalization procedures for oligonucleotide array data based on spiked cRNA controls. Genome Biol.

[B3] Lee PD, Sladek R, Greenwood CM, Hudson TJ (2002). Control genes and variability: absence of ubiquitous reference transcripts in diverse mammalian expression studies. Genome Res.

[B4] van de Peppel J, Kemmeren P, van Bakel H, Radonjic M, van Leenen D, Holstege FC (2003). Monitoring global messenger RNA changes in externally controlled microarray experiments. EMBO Rep.

[B5] Yang YH, Dudoit S, Luu P, Lin DM, Peng W, Ngai J, Speed TP (2002). Normalization for cDNA microarray data: a robust composite method addressing single and multiple slide systematic variation. Nucleic Acids Res.

[B6] Hekstra D, Taussig AR, Magnasco M, Naef F (2003). Absolute mRNA concentrations from sequence-specific calibration of oligonucleotide arrays. Nucleic Acids Res.

[B7] Sterrenburg E, Turk R, Boer JM, van Ommen GB, den Dunnen JT (2002). A common reference for cDNA microarray hybridizations. Nucleic Acids Res.

[B8] Dudley AM, Aach J, Steffen MA, Church GM (2002). Measuring absolute expression with microarrays with a calibrated reference sample and an extended signal intensity range. Proc Natl Acad Sci USA.

[B9] Talaat AM, Howard ST, Hale W, Lyons R, Gamer H, Johnston ST (2002). Genomic DNA standards for gene expression profiling in Mycobacterium tuberculosis. Nucleic Acids Res.

[B10] Bolstad BM, Irizarry RA, Astrand M, Speed TP (2003). A comparison of normalization methods for high density oligonucleotide array data based on variance and bias. Bioinformatics.

[B11] Lockhart DJ, Dong H, Byrne MC, Follettie MT, Gallo MV, Chee MS, Mittmann M, Wang C, Kobayashi M, Horton H, Brown EL (1996). Expression monitoring by hybridization to high-density oligonucleotide arrays. Nat-Biotechnol.

[B12] Kanno J, Onyon L, Peddada S, Ashby J, Jacob E, Owens W (2003). The OECD program to validate the rat uterotrophic bioassay. Phase 2: dose-response studies. Environ Health Perspect.

[B13] Kanno J, Inoue T, Pennie ED (2002). Reverse toxicology as a future predictive toxicology. Toxicogenomics.

